# Outer membrane vesicles blebbing contributes to *B. vulgatus* mpk-mediated immune response silencing

**DOI:** 10.1080/19490976.2017.1344810

**Published:** 2017-07-13

**Authors:** Jan Kevin Maerz, Alex Steimle, Anna Lange, Annika Bender, Birgit Fehrenbacher, Julia-Stefanie Frick

**Affiliations:** a Institute of Medical Microbiology and Hygiene, University of Tübingen, Tübingen, Germany; b University Department of Dermatology, University of Tübingen, Tübingen, Germany

**Keywords:** Bacteroides vulgatus, dendritic cells, immune response, outer membrane vesicles

## Abstract

The Gram negative intestinal symbiont *Bacteroides vulgatus* mpk is able to prevent from induction of colonic inflammation in *Rag1^−/−^* mice and promotes immune balance in *Il2^−/−^* mice. These inflammation-silencing effects are associated with *B. vulgatus* mpk-mediated induction of semi-mature dendritic cells, especially in the colonic lamina propria (cLP). However the beneficial interaction of bacteria with host immune cells is limited due to the existence of a large mucus layer covering the intestinal epithelium. How can intestinal bacteria overcome this physical barrier and contact the host immune system?

One mechanism is the production of outer membrane vesicles (OMVs) via ubiquitous blebbing of the outer membrane. These proteoliposomes have the ability to traverse the mucus layer. Hence, OMVs play an important role in immunomodulation and the maintenance of a balanced gut microbiota. Here we demonstrate that the stimulation of bone marrow derived dendritic cells (BMDCs) with isolated OMVs originated from *B. vulgatus* mpk leads to the induction of a tolerant semi-mature phenotype. Thereby, microbe- associated molecular patterns (MAMPs) delivered by OMVs are crucial for the interaction and the resulting maturation of immune cells. Additional to the binding to host TLR4, a yet unknown ligand to TLR2 is indispensable for the conversion of immature BMDCs into a semi-mature state. Thus, crossing the epithelial mucus layer and directly contact host cells, OMV mediate cross-tolerance via the transport of various Toll-like receptor antigens. These features make OMVs to a key attribute of *B. vulgatus* mpk for a vigorous acellular prevention and treatment of systemic diseases.

## Introduction

The intestinal microbiota provides important features which are considered to be beneficial for the host organism, such as (1) the maintenance of the structural integrity of the intestinal mucosal barrier, (2) the development of epithelial cell function, (3) host nutrient metabolism, (4) modulation of the immune system and (5) prevention of bacterial overgrowth.^1,2^ Hereby, a certain microbial diversity is considered to be required for the maintenance of the immune homeostasis and crucial for the optimal functionality and interaction with the host. Concerning the interplay between microbiota and the host immune system, a disturbed or altered composition of the gut microbiota, called “dysbiosis,” is associated with the pathogenesis of both intestinal and systemic immunological disorders.^3^ Such a dysbiosis of the intestinal microbiota, can also influence the disease onset and progress in a variety of mouse models for autoimmune diseases (AID) such as rheumatoid arthritis (RA), type 1 diabetes (T1D) and inflammatory bowel disease (IBD).^4-6^ This highlights the importance of a deeper knowledge of the interaction between the host immune system and the intestinal microbes. Based on this cross talk, specific modulation of the intestinal microbiota composition is considered to be used as therapy for the treatment of microbiota-associated AID.^7,8^ Hereby, intestinal commensals are decisive for the induction (pathobionts) or prevention (symbionts) of a pathological immune response in a certain pre-disposed host.^9^ In this context, members of the genus *Bacteroides* were already demonstrated to exhibit symbiotic properties concerning the modulation of the intestinal immune system.^10^

Accordingly, *Bacteroides vulgatus* mpk was already depicted to mediate inflammation-silencing effects finally preventing from colitis induction in various moue models for IBD.^1-13^ These immune regulatory properties of *B. vulgatus* mpk are traced back to the induction of a tolerant dendritic cell (DC) phenotype in the colonic lamina propria (cLP) termed semi-mature.^12-14^ However, direct physical interaction between intestinal commensals and cLP DCs is limited due to the presence of a thick and almost sterile mucus layer covering the intestinal epithelium. Therefore, we were interested in how this symbiotic commensal manages to enhance the communication with host cells to modulate the immune system.

Instead of direct physically interacting with target cells, bacteria can communicate with more distant host environments via secretion of signaling molecules, such as toxins, quorum sensing molecules and DNA.^15^ These mechanisms involving non-viable bacteria provide distinct advantages for bacteria, since secreted soluble material is smaller and non-viable.^16,17^ Therefore, this material can gain access to environments usually being inaccessible for living bacteria. In this context, the biogenesis and release of bacterial outer membrane vesicles (OMVs) by Gram-negative bacteria represent a secretion pathway combining the long-distance traveling ability of small soluble bacterial products with mimicking the characteristics of the whole bacterial cell by facilitating the transport of insoluble molecules.^18,19^ OMV release therefore constitutes a critical mechanism for intra- and inter-kingdom communication.^20,21^ Derived from the outer membrane of bacteria, OMVs harbour a broad variety of microbe-associated molecular patterns (MAMPs) which interact with pattern recognition receptors (PRRs) of host target cells, leading to innate and adaptive immune responses.^22,23^ It was shown that the application of OMVs critically modulates the course of disease in animal models of intestinal inflammation by either a direct or an indirect interaction with the intestinal immune system.^24,25^ Hickey et al. even showed that OMVs of *B. thetaiotaomicron* can be found in macrophages of the intestinal mucosa. This implicates a passaging through not only the mucus layer but also through the intestinal epithelial cell layer.

In this study, we provide evidence that the symbiotic commensal *B. vulgatus* mpk produces OMVs. This generation of OMVs contribute to the mediation of the immune-system silencing properties provoked by this strain via the induction of DC semi-maturation. Thereby, both TLR2- and MD-2/TLR4-mediated signaling is crucial for this OMV-induced generation of semi-mature DCs, indicating that TLR2 and TLR4 ligands carried by OMVs are indispensable for the manifestation of this tolerant DC phenotype. Therefore, B. vulgatus mpk-caused OMV blebbing seems to contribute to host-microbe communication at intestinal sites which are hardly accessible for the living bacterium.

## Results

### Bacteroides vulgatus mpk outer membrane vesicles induce tolerance in CD11c^+^ cells

It is well known that components of the intestinal microbiota have widespread effects on the mucosal immune system in the intestine.^14,26^ Hereby, commensal bacteria exhibit important functions for the priming of immune cells underlying the mucosal epithelial barrier. However, physical contact between bacteria and such immune cells is restricted due to the presence of a large mucus layer covering the intestinal epithelium. Almost all bacteria produce outer membrane vesicles (OMVs) through bulging of the outer bacterial membrane.^27^ These vesicles are able to cross the mucin layer, finally increasing the probability of interaction of mucosal immune cells with bacterial surface structures such as MAMPs.^28^ The mechanism of OMV production is a key characteristic of Gram negative bacteria and commensal-derived OMVs seem to be important for the modulation of the host immune system.^29^

**Figure 1.**
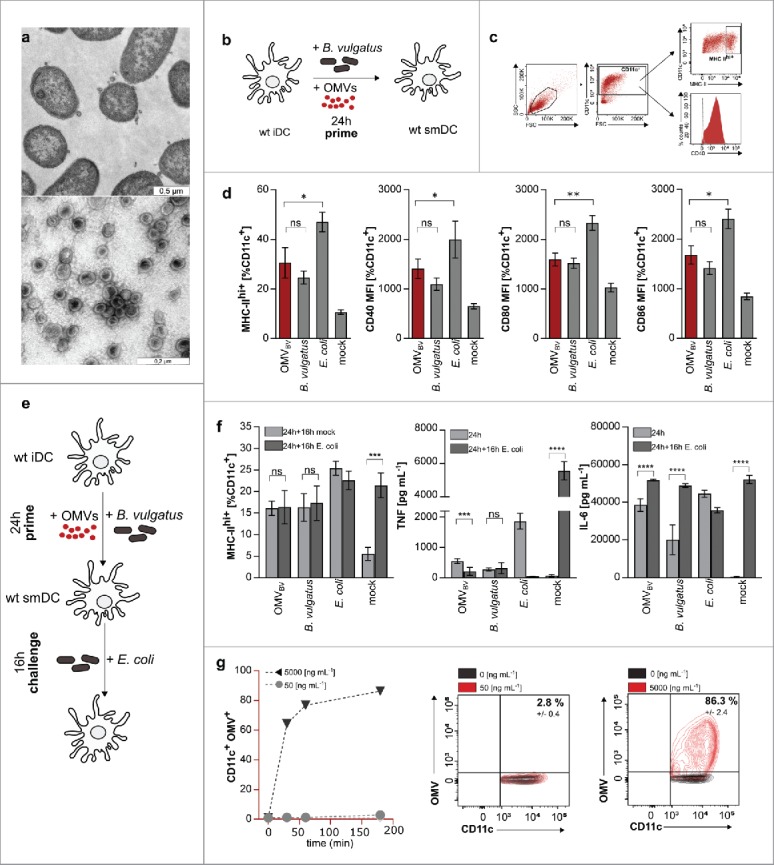
Induction of dendritic cell semi-maturation via *B. vulgatus* OMVs (OMV_BV_). (a) Evidence for vesicle production derived from the outer membrane of cultivated *Bacteroides vulgatus* cell. Black arrows indicate single secreted vesicles of fixed bacterial cells after toluidine blue staining captured with Transmission Electron Microscope (upper picture). Native OMV_BV_ after isolation and purification stained negative with uranyl acetate (lower picture). (b) Experimental setting for the analysis of surface maturation marker expression on dendritic cells after stimulation with OMVs. BMDCs are primed for 24 h with either PBS (mock), *B. vulgatus* or *E. coli* as control and additionally with 50 ng mL^−1^ OMV_BV_. (c) Gating strategy for the determination of MHC class II high positive (MHC-II ^hi+^), CD40, CD80 or CD86 BMDCs. CD11c-negative cells were excluded and the proportion of MHC-II ^hi+^ (dot plot), CD40, CD80 or CD86 (histogram) DCs within the population of CD11c^+^ cells was determined as shown. Dashed line shows negative control. (d) MHC class II ^hi+^, CD40, CD80 or CD86 population CD11c^+^ BMDCs that were primed with PBS (mock), *B. vulgatus* mpk, 50 ng mL^−1^ OMV_BV_ or *E. coli* mpk for 24 h (n = 4). (e) Experimental setting for investigation of dendritic cell tolerance after induction of semi-maturation. BMDCs are primed for 24 h with either PBS (mock) to preserve an immature phenotype, *B. vulgatus* mpk or 50 ng mL^−1^ OMV_BV_ to induce semi-maturation or *E. coli* to induce BMDC maturation. After medium change, these cells were secondarily challenged for 16 h with either PBS (mock) as controls or *E. coli* to proof non-responsiveness of tolerant cells. (f) Expression of analyzed maturation markers MHC class II, TNF and IL-6 of CD11c^+^ BMDCs after priming with PBS (mock), *B. vulgatus* mpk, 50 ng mL^−1^ OMV_BV_ or *E. coli* mpk for 16 h and subsequently challenge with either PBS (mock) or *E. coli* mpk for 16 h (n = 4). (g) Flow cytometry analysis of OMV_BV_ internalization by BMDCs. Different concentrations of vesicles were labeled with fluorescein isothiocyanate (FITC) and incubated with cultured DCs for various times. Percentages show CD11c^+^OMV_BV_^+^ cell populations. Unlabeled OMVs served as negative control (black population in dot plot graph). All statistical analyses were performed using student's t test. Error bars represent SD.

In previous work, we could demonstrate that *B. vulgatus* mpk modulate the immune response *in vivo* and *in vitro* in an inflammation-silencing manner. This is mainly mediated by the induction of tolerant CD11c^+^ cells in the colonic lamina propria, which seem to be responsible for the maintenance of homeostatic conditions in the intestine.^12,13^ Several molecular mechanisms for tolerance induction have already been identified in CD11c^+^ bone marrow derived dendritic cells (BMDCs).^30^ Until now, not all immune system-modulating surface structures of *B. vulgatus* mpk were already identified. Therefore, we were interested if this symbiotic commensal produces OMVs and if this vesicle production contributes to the observed immuno-modulatory properties of this commensal strain. The secretion of OMVs can be a response due to environmental stress of the prokaryotic cell.^28,31^ Therefore, we incubated the bacteria under different culture conditions and for different periods of time, to elucidate, if this strain produces OMVs and if this is dependent on the culturing conditions. We could demonstrate that *B. vulgatus* produces vesicles ubiquitarily under non-detrimental conditions through bulging of the outer membrane as demonstrated in TEM pictures (Fig. 1a). To gain information on the immune system modulating features of OMVs, CD11c^+^ BMDCs were generated as described and stimulated with *B. vulgatus* mpk-derived OMVs (OMV_BV_) for 24 h (Fig. 1b). After end of incubation time, BMDCs were checked for surface expression of MHC-II, CD40, CD80 and CD86 by flow cytometry (Fig. 1c, gating strategy) since these surface markers indicate the maturation status of CD11c^+^ BMDCs.^11,12,14,32^ PBS-stimulated BMDCs were used as a negative control (mock), representing completely immature CD11c^+^ cells. BMDCs stimulated with pathobiotic *E. coli* mpk were used as positive controls, representing mature CD11c^+^ cells, since CD11c^+^ BMDC stimulation with this strain was demonstrated to induce complete maturation.^11^ *B. vulgatus* mpk was already demonstrated to induce CD11c^+^ cell semi-maturation as indicated by intermediate expression of MHC-II, CD40, CD80 and CD86 which was significantly lower compared with the expression of these respective surface proteins of mature CD11c^+^ BMDCs.^11^ We could demonstrate that OMV_BV_ provide the same properties like live *B. vulgatus* mpk when used at a concentration of 50 ng mL^−1^, since we could not detect any difference in the expression of MHC-II, CD40, CD80 and CD86 between CD11c^+^ cells stimulated with either OMV_BV_ or live *B. vulgatus* mpk, respectively (Fig. 1d). Additionally and as confirmed for live *B. vulgatus* mpk, CD11c^+^ cells stimulated with OMV_BV_ provided significantly lower surface expression of these proteins compared with *E. coli* mpk stimulated CD11c^+^ BMDCs. However, the surface expression of MHC-II and T cell co-stimulatory surface proteins of CD11c^+^ BMDCs represents only a first hint on the maturation of these cells. A key feature of semi-mature CD11c^+^ cells is tolerance toward a secondary bacterial stimulus after priming with *B. vulgatus* mpk.^11^ Therefore, we primed CD11c^+^ BMDCs with either OMV_BV_, live *B. vulgatus* mpk, *E. coli* mpk or PBS (mock), incubated these cells for 24 h, changed cell culture medium and challenged them with either PBS or *E. coli* mpk (Fig. 1e). We analyzed the surface expression of MHC-II and the secretion of the pro-inflammatory cytokines TNF and IL-6. Immature PBS-stimulated CD11c^+^ cells showed a significantly increased surface expression of MHC-II as well as secretion of TNF and IL-6 after challenge with *E. coli* mpk. Already mature *E. coli* mpk primed CD11c^+^ cells maintained their high expression level of MHC-II and IL-6 after challenge with *E. coli* mpk. Due to the high secretion of TNF during the first 24 h, indicating a maturation of the BMDCs, secreted TNF was not detectable in the supernatant of mature CD11c^+^ cells upon a second challenge with *E.coli* mpk after exchange of media.^33,34^ This is in line with the literature reporting on a non-responsiveness of mature BMDCs to additional stimuli.^11,32,35^ Semi-mature CD11c^+^ cells stimulated with either live *B. vulgatus* mpk or OMV_BV_ were non-responsive toward subsequent challenge with *E. coli* mpk concerning the surface expression of MHC-II and the secretion of TNF in the supernatant (Fig. 1f). Furthermore, to provide evidence for a direct interaction of *B. vulgatus*-derived OMVs with antigen-presenting cells, we performed an internalisation timeline assay using CD11c^+^ BMDCs in combination with different concentration of FITC-labeled vesicles. OMV_BV_ were taken up by cultured BMDCs, leading to an extensive increase of CD11c^+^OMV_BV_^+^ cell population after 30 minutes as shown in Figure 1g. As shown by Shen et al., the rapid internalization of vesicles can be achieved in an actin-dependent manner directing the expression of surface markers and inflammatory cytokine secretion in BMDCs.^24^

### OMV_BV_-mediated immunomodulatory properties are sensed via host TLR2 and TLR4 receptor complexes

**Figure 2.**
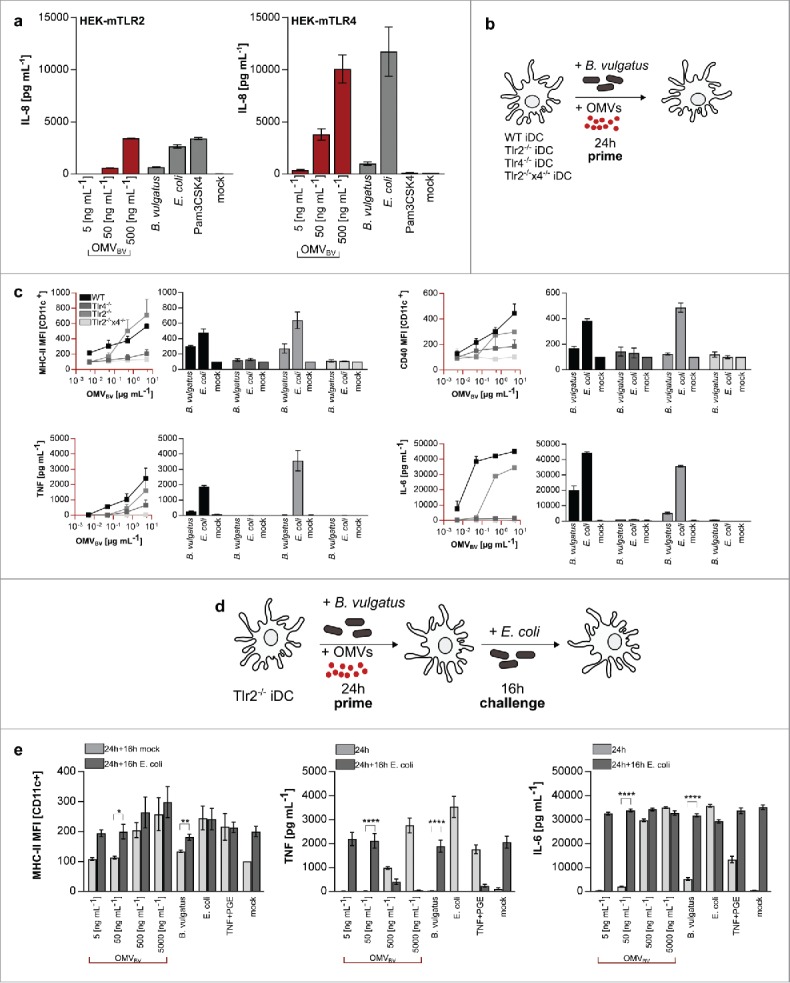
Induction of dendritic cell tolerance via OMV_BV_ is TLR2 and TLR4 dependent. (a) Stimulation of Human embryonic kidney (HEK) cells overexpressing murine TLR 2 or 4. HEK cells are primed with PBS (mock), *B. vulgatus* mpk, various concentrations of OMV_BV_ or *E. coli* mpk for 24 h. Pam3CSK4 served as TLR2 specific control. (b) Experimental setting for the analysis of surface maturation marker expression on dendritic cells after stimulation with OMVs. *wt, Tlr2^−/−^, Tlr4^−/−^* and *Tlr2^−/−^xTlr4^−/−^* BMDCs are primed for 24 h with either PBS (mock), *B. vulgatus* or *E. coli* as control and additionally with increasing concentrations of OMV_BV_. (c) MHC class II^hi+^ or CD40 population (CD11c^+^) (normalized to mock) and secreted concentration of TNF or IL-6 of *wt* and TLR-deficient BMDCs that were primed with PBS (mock), *B. vulgatus* mpk, different concentrations of OMV_BV_ or *E. coli* mpk for 24 h (n = 4). (d) Experimental setting for investigation of dendritic cell tolerance after induction of semi-maturation in Tlr2^−/−^ DCs.BMDCs are primed for 24 h with either PBS (mock) to preserve an immature phenotype, *B. vulgatus* or OMV_BV_ to induce semi-maturation or *E. coli* to induce BMDC maturation. After medium change, these cells were secondarily challenged for 16 h with either PBS (mock) as controls or *E. coli* to proof non-responsiveness of tolerant cells. (e) Expression of analyzed maturation markers MHC class II, TNF and IL-6 of CD11c^+^ BMDCs of TLR2^−/−^ mice after priming with PBS (mock), *B. vulgatus* mpk, OMV_BV_ or *E. coli* mpk for 16 h and subsequently challenge with either PBS (mock) or *E. coli* mpk for 16 h (n = 4). All statistical analyses were performed using student's t test. Error bars represent SD.

We demonstrated that *B. vulgatus* mpk and its outer membrane vesicles interact with innate immune cells and modulate the host immune system in an anti-inflammatory sense. Though, the exact mechanisms and involved pattern recognition receptors for signal transduction were not yet accurately identified. Therefore, we wanted to describe the responsible receptor on host target cells for the interaction with microbial ligands on bacterial cell surface and outer membrane vesicles, respectively. In this context, Toll-like receptors (TLRs) are the most pivotal pattern-recognition receptors (PRRs) for the detection of MAMPs.^36-39^ The most abundant MAMPs exhibiting strong immunogenic properties are lipopolysaccharides (LPS), which are recognized by CD14 and the TLR4/MD-2 receptor complex.^14,40-42^ Besides TLR4 agonists, TLR2 ligands like bacterial lipopeptides, play a prominent role regarding target activation of antigen presenting cells.^43-45^ In general, TLR4- as well as TLR2- agonists can be integrated into OMVs and therefore be transported by these vesicles.^46^ To characterize the *B. vulgatus* mpk- and OMV_BV_- associated ligands which might be relevant for the induction of semi-mature BMDCs, we used Human Embryonic Kidney cells (HEK) overexpressing mouse TLR2 or the mouse CD14/TLR4/MD-2 receptor complex. The resulting IL-8 secretion into cell supernatant was detected to determine receptor activation. PBS treated cells served as negative control, whereas Pam_3_CSK_4_ was used as TLR2 ligand. As demonstrated in Fig. 2a, OMV_BV_ induced both activation of the TLR2 receptor as well as CD14/TLR4/MD-2 receptor complex. To validate these results, we used CD11c^+^ BMDCs derived from mice deficient for Toll-like receptor 2 (*Tlr2^−/−^*), TLR4 (*Tlr4^−/−^*) and for both receptors (*Tlr2^−/−^xTlr4^−/−^*). CD11c^+^ BMDCs of *wt* and *Tlr2^−/−^, Tlr4^−/−^* and *Tlr2^−/−^xTlr4^−/−^* mice were generated as described and primed with OMV_BV_ for 24 h (Fig. 2b). CD11c^+^ cells were harvested and analyzed for surface expression of MHC-II and CD40 (Fig. 1c, gating strategy). Additionally, the amount of secreted pro-inflammatory TNF and IL-6 was detected. The deficiency of both TLRs on the cell surface leads to a complete loss of antigen recognition independent of the used stimulus as indicated by the low expression of MHC-II and an abolished secretion of TNF of *Tlr2^−/−^xTLR4^−/−^* CD11c^+^ BMDCs. In accordance with the findings generated with HEK cells (Fig. 2a), the activation of the TLR4/MD-2 receptor complex via *B. vulgatus* mpk- secreted OMVs is decisive for the main outcoming signal. In addition, the expression of MHC-II, the costimulatory proteins and the secretion of the inflammatory cytokines in *Tlr2−/−* stimulated BMDCs is nearly as strong as in the *wt* cells after challenging with *E.coli* mpk, *B. vulgatus* mpk or high concentration of OMV_BV_. Nevertheless, we also observed a dose-dependent increase of all analyzed proteins upon stimulation of *Tlr4^−/−^* CD11c^+^ BMDCs with purified OMV_BV_, pointing out that the TLR2 receptor is competent to recognize agonistic structures present in OMV_BV_. Furthermore, TLR2-dependent signaling is also involved in vesicle- induced BMDC activation and maturation (Fig. 2c). This observation prompted us to elucidate whether the contact of host immune cells with a bacterial TLR4 agonist such as LPS alone is sufficient for the activation and differentiation of tolerant BMDCs or whether an additional TLR2 activation is accessory required for adequate induction of BMDC semi-maturation. Therefore, BMDCs were generated from *Tlr2^−/−^* mice, primed with OMV_BV_ for 24 h and challenged with *E. coli* mpk for additional 16 h (Fig. 2d). As mentioned before, this experimental setting is required to check for tolerance in semi-mature BMDCs. We could verify that a TLR2-dependent signaling is necessary for tolerance induction in BMDCs, since *Tlr2^−/−^* failed to become tolerant upon priming with *B. vulgatus* mpk or OMV_BV_ as demonstrated by the high expression of MHC class II and the enhanced secretion of TNFα upon *E. coli* mpk challenge (Fig. 2e).

## Discussion

In this study, we demonstrated that the Gram negative symbiotic commensal *B. vulgatus* mpk produces outer membrane vesicles (OMV). This OMV blebbing mechanism seems to contribute to the immune response modulating properties of this strain which was demonstrated in several previous studies. *B. vulgatus* mpk-derived OMVs (OMV_BV_) induced tolerance in CD11c^+^ BMDCs in a TLR4 and TLR2 dependent manner. However, the defined vesicle-associated receptor ligands were not identified. Nevertheless, simultaneous activation of both receptors was required to induce effective DC tolerance. In line with Shen et al. and Hickey et al.^24,25,27^ this feature indicates an additional mechanism how *B. vulgatus* mpk as a symbiotic commensal, modulates the immune system at mucosal interfaces. As Bacteria are separated from the host intestinal mucosa by a thick mucus layer that is thought to impede a direct contact between luminal bacteria and the intestinal mucosa,^47^ the shedding of OMVs by *B. vulgatus* is of special importance since OMVs can diffuse through the mucin layer.^25,48^ This underlines the role of OMVs for the modulation of intestinal immune cells. These characteristics make OMV_BV_ to an important attribute of *B. vulgatus* mpk for an effective passive protection and treatment of systemic diseases.

Gram negative bacteria are able to deliver a chemically diverse spectrum of cargo protected from the external environment over long distances by the help of outer membrane vesicles.^49^ Though, OMVs can cross physical barriers which are impermeable for the whole bacterium such as the intestinal mucus layer, gain access to the intestinal epithelium and therefore to the underlying immune cells present in the intestinal lamina propria.^24,25,28,48^ This makes OMV blebbing to an important immune modulating mechanism. Considering pathogens, vesicles of *Salmonella enterica* or *Helicobacter pylori* harbour virulence factors to enhance bacterial survival and the ability to colonize host mucosal tissues.^50,51^ This leads to a stringent immune reaction and local inflammation.^52^ However, OMVs derived from commensal symbiotic bacteria were demonstrated to be involved in maintaining or restoring immune homeostasis in the intestine.^53,54^ For example, *Bacteroides fragilis*, representing an intestinal commensal, produces OMVs and interacts with the host immune system by delivering a capsular polysaccharide (PSA) to dendritic cells.^21,55^ The release of PSA in OMVs induces immune-modulatory effects, activates regulatory T cells and prevents from experimental colitis.^24^ However, a general role for OMVs in mediating immune responses, accounting for all OMV-producing commensal, was not yet demonstrated.^31,56^

Findings using live *B. vulgatus* mpk cells indicate that this symbiotic bacterium prevents from disease induction in different mouse models for experimental colitis.^12,13^ In this context, *B. vulgatus* mpk promotes the maintenance of the immune equilibrium via induction of tolerant semi-mature dendritic cells in the intestine and the resulting regulation of host Cathepsin S activity and secretion in these antigen-presenting cells.^32^ In this study, we demonstrated that OMVs derived from *B. vulgatus* mpk contribute to these observed effects since they interact with bone marrow derived dendritic cells, leading to a semi-mature phenotype characterized by a low expression of T cell activation and maturation markers as well as pro-inflammatory cytokines.

Innate immune cells recognize conserved microbial ligands through pattern recognition receptors (PRRs) leading to immunologic responses. For example, Polysaccharide A (PSA) from *Bacteroides fragilis* is recognized by TLR2. In consequence, the binding of PSA to TLR2 leads to the induction of FoxP3^+^ regulatory T cells which affect both the development and homeostasis of the host immune system.^57,58^ Our study now reveals that OMV_BV_ also contain TLR2 activating ligands which are, in consequence, necessary for the induction of tolerant BMDCs. Since these tolerance-inducing effects are abolished in TLR2- deficient BMDCs, a TLR2-dependent intracellular signaling is indispensable for OMV_BV_-mediated induction of tolerance in BMDCs. These observations indicate that both receptors, MD-2/TLR4 and TLR2, are required for the induction of DC tolerance. In addition to the LPS in the outer membrane, OMVs of *B. vulgatus* mpk contain and a yet unidentified TLR2 agonist, as it is e.g. the case for PSA in vesicles secreted by *B. fragilis*. Interestingly, the genome of *B. vulgatus* mpk contains 9 different loci coding for capsular polysaccharides (e.g., glycosyltransferases and polysaccharide export outer membrane proteins), indicating the presence of a TLR2-interacting polysaccharide on the outer membrane of the bacterium cell and on vesicles derived from *B. vulgatus* mpk.^46^ But the comparison of the various loci in *B. fragilis* and *B. vulgatus* revealed different arrangements of the genes necessary for the biosynthesis of capsular polysaccharides (data not shown). This implicates, that polysaccharides present in *B. vulgatus* mpk has a different structure as compared with the capsular polysaccharide produce by *B. fragilis*.

The presence of a TLR4- and TLR2- ligand on the outer membrane indicates that the mechanism by which OMV_BV_ induce tolerance in DCs is not originated in classical endotoxin tolerance. Usually, low doses of one TLR agonist desensitize the host to subsequent stimulation with a related agonist or endotoxin.^59-61^ However, the addition of a Toll-like receptor (TLR) ligand can also induces tolerance to subsequent challenges with other stimuli that signal through one or more different TLRs (cross-tolerance). Since outer membrane vesicles harbor various different TLR ligands, OMV_BV_ might even mediate TLR-cross-tolerance. The simultaneous interaction of different MAMPs with different receptors might therefore result in complementary, synergistic or antagonistic effects that modulate innate and adaptive immunity.^62-64^

In summary, outer membrane vesicles derived from the symbiotic intestinal commensal of *B. vulgatus* mpk play a pivotal role in modulating and regulating immune responses of the host. With their combined qualities of crossing physically barriers and the mediation of endotoxin tolerance and even cross-tolerance in dendritic cells via the delivery of different microbial ligands to immune cells, the production of OMV represents an important key feature of this symbiotic strain, to prevent from intestinal inflammation in the host. Due to these properties, OMV_BV_ might even represent a potential therapeutical tool to hamper or recover from inflammatory bowel diseases (IBD) and other systemic inflammatory disorders.

## Materials and methods

### Cultivation of bone marrow derived dendritic cells (BMDCs)

Bone marrow cells were isolated and cultivated for differentiation as described previously.^65^ At day 7 after isolation, resulting CD11c positive, bone marrow derived dendritic cells (BMDCs) were harvested and used for stimulation.

### Cultivation of human embryonic kidney cells (HEK cells)

Before stimulation, cryoconserved cell stocks were thawed and cultured in DMEM media supplemented with 10% FCS, 1% Penicillin/Streptomycin up to passage 7.

### Stimulation of bone marrow derived dendritic cells

Two × 10^6^ BMDCs were stimulated with either PBS or *B. vulgatus* mpk or *E. coli* mpk at a MOI of 1 or various concentrations of *B. vulgatus*-derived OMVs (5, 50, 500, 5000 ng mL^−1^). Cells were stimulated for a maximum of 24 hours. If a second challenge was used, media were changed and cells were restimulated with either PBS or *E. coli* mpk MOI 1 for a maximum of 16 hours. Additionally, exogenous TGF (Sigma-Aldrich) 20 ng mL^−1^ and prostaglandin E (Sigma-Aldrich) 1 µM were used as positive and Toll-like receptor- independent control.

### Stimulation of human embryonic kidney cells

Two × 10^4^ HEK cells were stimulated with either PBS or *B. vulgatus* mpk or *E. coli* mpk at a MOI of 1 or various concentrations of *B. vulgatus*-derived OMVs (5, 50, 500 ng mL^−1^). Cells were stimulated for a maximum of 24 hours. FSL-1 (10, 100 ng mL^−1^) and Pam3CSK4 (30, 300 ng mL^−1^) (both InvivoGen) were used as positive and TLR2-specific agonist.

### Flow cytometrical analysis

Multi-color FCM analyses were performed on a FACS LSRII (BD Biosciences). All fluorochrome-coupled antibodies were purchased from BD Biosciences. Data were analyzed using the FlowJo software (Tree Star Inc., USA).

### Cytokine analysis by ELISA

For analysis of IL6, TNF and IL1ß concentrations in cell culture supernatants, ELISA Kits purchased from BD Bioscience were used according to the manufacturer's instructions.

### Bacteria cultivation

The bacteria used for stimulation of the murine dendritic cells were *Escherichia coli* mpk and *Bacteroides vulgatus* mpk. The *E. coli* mpk strain was grown in Luria-Bertani (LB) medium under aerobic conditions at 37°C. *Bacteroides vulgatus* was grown in Brain-Heart-Infusion (BHI) medium and anaerobic conditions at 37°C.

### Detection of secreted OMVs

Part of the cultured bacteria suspension was sedimented for 2 h and fixed with Paraformaldehyde-Glutaraldehyde Solution (Karnovsky's Fixative) in a rate of 7:2 (A:B) for 1 h at room temperature after removal of excess fluids. Following, for the stabilization of proteins and lipids samples were post-fixed in osmium tetroxide. Fixed cells are then embedded in agar and washed in 0.1 M cacodylate buffer. Addition of uranyl acetate for 1 h enhanced membrane stabilization and improved overall contrast. Dehydration was performed with an ETOH dilution row, followed by transfer in epoxide solution. The polymerization of the agarose pad was achieved via incubation for 20 h at 45°C and plus 48 h at 60°C. For visualization, the semi-thin sections were treated with toluidine blue staining. Images were recorded with Zeiss Libra 120 Transmission Electron Microscope.

### Isolation protocol and microscopy of purified OMVs

Cultured *B. vulgatus* mpk suspension was slightly vortexed and centrifuged for 10 min at 10,000 x g. Supernatant was vacuum sterile filtrated and vesicles were pelletized for 2 h at 38,000 x g. Pellets were resuspended in sterile PBS and mixed with Iodixanol (OptiPrep) density gradient solution to prepare a 40% working solution. Subsequently, a dilutions serial with decreasing densities of 40%, 35%, 30%, 25%, 20% and 5% OptiPrep was assembled and stacked up in an appropriate ultracentrifugation tube. The samples were fractionated for 3 h at 280,000 x g and the developed, vesicle containing layers were collected. The isolated vesicles were natively held via the grid-on-drop technic and stained for negative imaging via TEM with uranyl acetate.

### FITC-labeling of isolated vesicles

Isolated and purified OMVs were incubated in the dark for 1 h with 1 mg mL^−1^ Fluorescein isothiocyanate (FITC) at room temperature. Suspension was washed twice with HEPES buffer and centrifuged for 30 min at 52,000 x g. The pellet containing fluorescence-labeled OMVs was resuspended in sterile PBS.

### Determination of OMVs concentration for stimulation experiments

To ensure a standardized application of vesicles and invariant stimulation of BMDCs and HEK cells, the exact concentration of every OMV batch was calculated and defined by the Pierce™ BCA Protein Assay Kit using the manufacturer's instructions.

### Mice

C57BL/6J mice were purchased from Charles River Laboratories. Toll-like receptor 2 and 4 deficient (*Tlr2^−/−^, Tlr4^−/−^*) mice were provided by Jackson Laboratory. All animals were kept and bred under SPF conditions. For isolation of bone marrow, only female mice aged 6–12 weeks were used. Animal experiments were reviewed and approved by the responsible institutional review committee and the local authorities.

### Statistics

Statistical analyses were performed using the unpaired student's t test. For comparing *in vitro* results, samples were considered to be biologically independent if the samples were generated from BMDCs from different mice. Differences were considered to be statistically significant if p < 0.05. Error bars, if shown, represent ± standard deviation (SD).
